# General versus central adiposity as risk factors for cardiovascular-related outcomes in a high-risk population with type 2 diabetes: a post hoc analysis of the REWIND trial

**DOI:** 10.1186/s12933-023-01757-z

**Published:** 2023-03-10

**Authors:** Edward Franek, Prem Pais, Jan Basile, Claudia Nicolay, Sohini Raha, Ana Hickey, Nadia N. Ahmad, Manige Konig, Hong Kan, Hertzel C. Gerstein

**Affiliations:** 1 Mossakowski Medical Research Centre, Polish Academy of Sciences and Central Clinical Hospital MSWiA, Warsaw, Poland; 2 St John’s Research Institute, Bangalore, India; 3 Medical University of South Carolina, Ralph H. Johnson VA Medical Center, Charleston, SC USA; 4 Eli Lilly and Company, Indianapolis, IN USA; 5 Population Health Research Institute, McMaster University and Hamilton Health Sciences, Hamilton, ON Canada

**Keywords:** Obesity, Cardiovascular risk, Cardiovascular disease, BMI, Waist circumference, Waist-to-hip ratio, Type 2 diabetes

## Abstract

**Background:**

In clinical practice, anthropometric measures other than BMI are rarely assessed yet may be more predictive of cardiovascular (CV) risk. We analyzed the placebo group of the REWIND CV Outcomes Trial to compare several anthropometric measures as baseline risk factors for cardiovascular disease (CVD)-related outcomes in participants with type 2 diabetes (T2D).

**Methods:**

Data from the REWIND trial placebo group (N = 4952) were analyzed. All participants had T2D, age ≥ 50 years, had either a previous CV event or CV risk factors, and a BMI of ≥ 23 kg/m^2^. Cox proportional hazard models were used to investigate if BMI, waist-to-hip ratio (WHR), and waist circumference (WC) were significant risk factors for major adverse CV events (MACE)-3, CVD-related mortality, all-cause mortality, and heart failure (HF) requiring hospitalization. Models were adjusted for age, sex, and additional baseline factors selected by LASSO method. Results are presented for one standard deviation increase of the respective anthropometric factor.

**Results:**

Participants in the placebo group experienced 663 MACE-3 events, 346 CVD-related deaths, 592 all-cause deaths, and 226 events of HF requiring hospitalization during the median follow-up of 5.4 years. WHR and WC, but not BMI, were identified as independent risk factors of MACE-3 (hazard ratio [HR] for WHR: 1.11 [95% CI 1.03 to 1.21]; p = 0.009; HR for WC: 1.12 [95% CI 1.02 to 1.22]; p = 0.012). WC adjusted for hip circumference (HC) showed the strongest association with MACE-3 compared to WHR, WC, or BMI unadjusted for each other (HR: 1.26 [95% CI 1.09 to 1.46]; p = 0.002). Results for CVD-related mortality and all-cause mortality were similar. WC and BMI were risk factors for HF requiring hospitalization, but not WHR or WC adjusted for HC (HR for WC: 1.34 [95% CI 1.16 to 1.54]; p < 0.001; HR for BMI: 1.33 [95% CI 1.17 to 1.50]; p < 0.001). No significant interaction with sex was observed.

**Conclusions:**

In this post hoc analysis of the REWIND placebo group, WHR, WC and/or WC adjusted for HC were risk factors for MACE-3, CVD-related mortality, and all-cause mortality; while BMI was only a risk factor for HF requiring hospitalization. These findings indicate the need for anthropometric measures that consider body fat distribution when assessing CV risk.

**Supplementary Information:**

The online version contains supplementary material available at 10.1186/s12933-023-01757-z.

## Background

Obesity, defined as excess adiposity that is detrimental to health, is a major risk factor for type 2 diabetes and other comorbidities [1]. Patients with type 2 diabetes and obesity have an increased risk for cardiovascular disease (CVD) [1]. Correspondingly, the American Diabetes Association recommends weight management strategies in addition to glycemic control for patients with type 2 diabetes [2].

Obesity can be assessed using different measures. In the absence of imaging modalities, which are typically not used in routine clinical practice, BMI, waist-to-hip ratio (WHR), and waist circumference (WC) are commonly used clinical measures. BMI can be readily calculated to estimate overall body fat, and WHR and WC can be measured during the office visit to estimate distribution of fat which may have varied pathophysiological effects. BMI measures weight to height squared ratio and is inclusive of total body fat and lean mass. WC and WHR measure central adiposity: WC examines the circumference of the abdomen at the level of the umbilicus, and WHR is a ratio of the circumference of the waist to that of the hips with higher ratios indicating more central adiposity [3].

Different measures of obesity have been associated with CVD and all-cause mortality. While some studies indicate that measures of central adiposity are superior to BMI when evaluating patients’ risk of cardiovascular (CV) events [4–6], the Emerging Risk Factors Collaboration showed no difference between BMI, WHR, and WC, in CVD risk prediction [7]. Similarly, while some studies suggest that WHR and WC are superior to BMI at predicting all-cause mortality risk [5], others indicate there is no difference between central and general adiposity measures [8–11]. Gender may also play a role in determining these relationships as WHR, but not BMI, was shown to independently predict major CV events (MACE) in female patients with coronary artery disease [12] and all-cause mortality in female patients with heart failure (HF) but not in male patients [13]. Superiority of BMI, WHR, or WC in predicting these events may also differ depending on the patient or population cohort.

The REWIND CV Outcomes Trial evaluated CVD-related events, including MACE-3, CVD-related mortality, all-cause mortality, and HF requiring hospitalization, over a median of 5.4 years [14, 15]. Patients had type 2 diabetes, were aged 50 years or older with CV risk factors or established CVD and had a baseline BMI of ≥ 23 kg/m^2^. The placebo group of the REWIND trial provides data on the CV outcomes of patients with type 2 diabetes being treated with the standard of care.

The aim of the current study was to evaluate and contrast measures of general and central adiposity as potential risk factors for MACE-3, CVD-related mortality, all-cause mortality, and HF requiring hospitalization in the placebo group of the REWIND CV Outcomes Trial.

## Methods

### Study design and patients

Data from the placebo group of the REWIND trial were used for this analysis. Details of the REWIND trial are published elsewhere [14, 15]. In brief, the REWIND trial was a global, multi-center, randomized, double-blind, placebo-controlled clinical trial. Participants with type 2 diabetes were aged ≥ 50 years with established CVD, aged ≥ 55 years with subclinical CVD, or aged ≥ 60 years with two or more CV risk factors. Participants (N = 9901) were randomized 1:1 to receive once-weekly subcutaneous injections of dulaglutide 1.5 mg or placebo in addition to the standard of care for diabetes and CVD of the specific country during the trial period of August 2011 to August 2018. Median follow-up was 5.4 years. All participants provided written and informed consent and the trial was conducted in accordance with the International Conference on Harmonization Guidelines for Good Clinical Practice and the Declaration of Helsinki.

Weight measurements were taken at baseline and throughout the trial annually as well as at the final study visit. Height, waist circumference, and hip circumference were measured at baseline and every 24 months throughout the trial as well as at the final study visit. To calculate BMI, body weight and height were measured. Body weight was measured using a calibrated scale (mechanical or digital). BMI was calculated as weight in kilograms divided by the square of height in meters. WC and hip circumference (HC) measurements were obtained with the patient in the standing position. WC was measured immediately above the iliac crest and HC at the maximal circumference of the buttocks, both in centimeters. WHR was calculated by dividing WC by HC.

The current analysis examined obesity measures, measured at baseline, as potential risk factors for four outcomes: MACE-3 (non-fatal myocardial infarction, non-fatal stroke, or death from CV causes including unknown causes), CVD-related mortality, all-cause mortality, and HF requiring hospitalization or urgent care. Potential CV outcomes and all deaths were adjudicated by an independent clinical endpoint committee that was masked to treatment assignment. Further adjudication criteria are published elsewhere [15].

### Statistical analyses

Analyses were conducted on all patients in the REWIND placebo group. Baseline demographic and other characteristics are summarized as means and standard deviations (SD) (continuous variables) and/or as counts and proportions (categorical variables).

Regression models were used to evaluate the relationship between three baseline measures of obesity (BMI, WHR and WC) and incident outcomes as described below. To account for the possibility that both WC and HC contain some prognostic information that may be lost by estimating a fixed WHR for each participant, WC was also included in a model that adjusted for the HC.

Results for the obesity measures were analyzed as hazard ratios (HR; 95% confidence intervals [CIs]) for one standard deviation (SD) increase. SD was 5.8 kg/m^2^ for BMI, 0.08 for WHR, 13.4 cm for WC, and 12.7 cm for HC.

Each obesity measure was assessed separately by first estimating its age and sex-adjusted hazard for each outcome with the Cox proportional hazards (CPH) regression model. If the respective obesity measure was a statistically significant risk factor for the corresponding outcome (p < 0.05) in this minimally adjusted model, the prespecified risk factors listed in Table 1 were added to this model, which was run using LASSO Cox regression to select significant variables [16]. This fully adjusted model was then scrutinized to determine whether the obesity measure continued to be a significant risk factor for the outcome.

**Table 1:** Baseline characteristics of the REWIND placebo group used as additional risk factors to adjust for obesity measures

	Placebo group (N = 4952)
Age (years)	66.2 (6.5)
Female	2283 (46.1)
Race	
American Indian or Alaska Native	543 (11.0)
Asian	218 (4.4)
Black or African American	346 (7.0)
Native Hawaiian or other Pacific Islander	22 (0.4)
White	3744 (75.6)
BMI (kg/m^2^)	32.3 (5.8)
BMI categories (kg/m^2^)	
Normal (< 25 kg/m^2^)	355 (7.2)
Overweight (25- < 30 kg/m^2^)	1566 (31.6)
Class I (30- < 35 kg/m^2^)	1640 (33.1)
Class II-III (≥ 35 kg/m^2^)	1391 (28.1)
Waist circumference (cm)	
Female	106.6 (13.0)
Male	110.8 (13.4)
Hip circumference (cm)	
Female	113.0 (13.6)
Male	108.5 (11.4)
Waist-to-hip ratio	
Female	0.95 (0.07)
Male	1.02 (0.07)
Baseline HbA1c (%)	7.4% (1.1)
Baseline eGFR (mL/min/1.73 m^2^)	
< 30	55 (1.1)
30–59	1063 (21.5)
60–89	2469 (49.9)
≥ 90	1238 (25.0)
Prior CVD^a^	1554 (31.4)
History of myocardial infarction	798 (16.1)
History of myocardial ischemia by a stress test or with cardiac imaging	447 (9.0)
Ischemic stroke	253 (5.1)
Coronary, carotid or peripheral artery revascularization	886 (17.9)
Unstable angina	306 (6.2)
Hospitalization for unstable angina with ECG changes	590 (11.9)
Systolic blood pressure (mm Hg)	137.3 (17.0)
Diastolic blood pressure (mm Hg)	78.5 (9.9)
UACR (mg/mmol)	1.9 (0.70–7.38)
Total cholesterol (mmol/L)	4.5 (1.16)
LDL cholesterol (mmol/L)	2.6 (0.98)
HDL cholesterol (mmol/L)	1.2 (0.36)
Non-HDL cholesterol (mmol/L)	3.3 (1.11)
Triglycerides (mmol/L)	1.60 (1.20–2.25)
Current tobacco usage	713 (14.4)
Past tobacco usage	2409 (48.6)
Current alcohol consumption	1736 (35.1)
Antihypertensive agents	4654 (94.0)
Lipid lowering agents	3485 (70.4)
Anticoagulant agents	2557 (51.6)
Antithrombotic agents	2909 (58.7)

Data are presented as mean (SD), n (%), or median (IQR)

*BMI* body mass index, *CVD* cardiovascular disease, *ECG* electrocardiogram, *eGFR* estimated glomerular filtration rate, *HDL* high-density lipoprotein, *IQR* interquartile range, *LDL* low-density lipoprotein, *N* number of patients in subgroup of population, *n* number of patients with stated characteristic, *SD* standard deviation, *UACR* urine albumin-to-creatinine ratio

^a^Prior CVD was defined as myocardial infarction, ischemic stroke, unstable angina with electrocardiogram changes, myocardial ischemia on imaging or stress test, or coronary, carotid, or peripheral revascularization

Two different combinations of three obesity measures (BMI, WHR, and WC or BMI, WC, and HC) were assessed together with multivariable CPH models, adjusted for age and sex. In contrast to the models described above, the three obesity measures were added to the pool of risk factors that underwent the variable selection process. As a result, final models could retain none to all three of the obesity measures. Analyses for the latter combination (BMI, WC, and HC) were repeated where HC was forced into the model to explore its impact on WC.

All final models were repeated with additional interaction factors for obesity measures and sex.

The proportional hazard assumption for the risk factors was checked visually as well as by testing whether their corresponding time dependent covariates were significant.

Collinearity of the four obesity measures (BMI, WHR, WC, and HC) was evaluated via calculating pairwise Pearson correlation coefficients and performing collinearity diagnostics following Belsley, Kuh, and Welsch [17]. WC was categorized into normal and obese, based on sex- and BMI-related thresholds [18], and cross-tabulated versus BMI categories.

Results from the multivariable CPH models are presented with HR and associated 95% CIs as well as p-values. For continuous risk factors including obesity measures, HRs are given for one SD increase.

All analyses presented are exploratory in nature, and a p value < 0.05 was considered statistically significant. Analyses were performed using SAS^©^ version 9.4., 2017 SAS Institute Inc., Cary, NC, USA.

## Results

### Baseline characteristics and demographics

There were 4952 participants in the REWIND placebo group. The average age was 66.2 years, 46.1% were female, and 75.6% were White (Table 1). At baseline, 31.4% had prior established CVD. Mean weight was 88.9 kg and BMI was 32.3 kg/m^2^. Mean WC was 110.8 cm for men and 106.6 cm for women. Mean HC was 108.5 cm for men and 113.0 cm for women and WHR was 1.02 for men and 0.95 for women.

### Incidence of health outcomes

During follow-up in the placebo group, there were 663 MACE-3 events, 346 CVD-related deaths, 592 all-cause deaths, and 226 events of HF requiring hospitalization or urgent care.

### Association of obesity measures with health outcomes

The list of variables included in the Stepwise Variable Selection can be found in Table 1 alongside the respective baseline values. There was a high correlation between BMI, HC, and WC (pairwise correlation coefficients: 0.78–0.83). WHR had a modest correlation with WC (correlation coefficient: 0.43), and only a minor or no apparent correlation with HC (correlation coefficient: -0.23) and BMI (correlation coefficient: 0.06). The majority of participants in the normal and obese WC categories fell within the overweight (25.7% and 34.6%, respectively), obesity Class I (37.5% and 30.9%, respectively), and obesity Class II BMI categories (32.6% and 25.9%, respectively) (Additional file 1: Fig. S1).

**Fig. 1:**
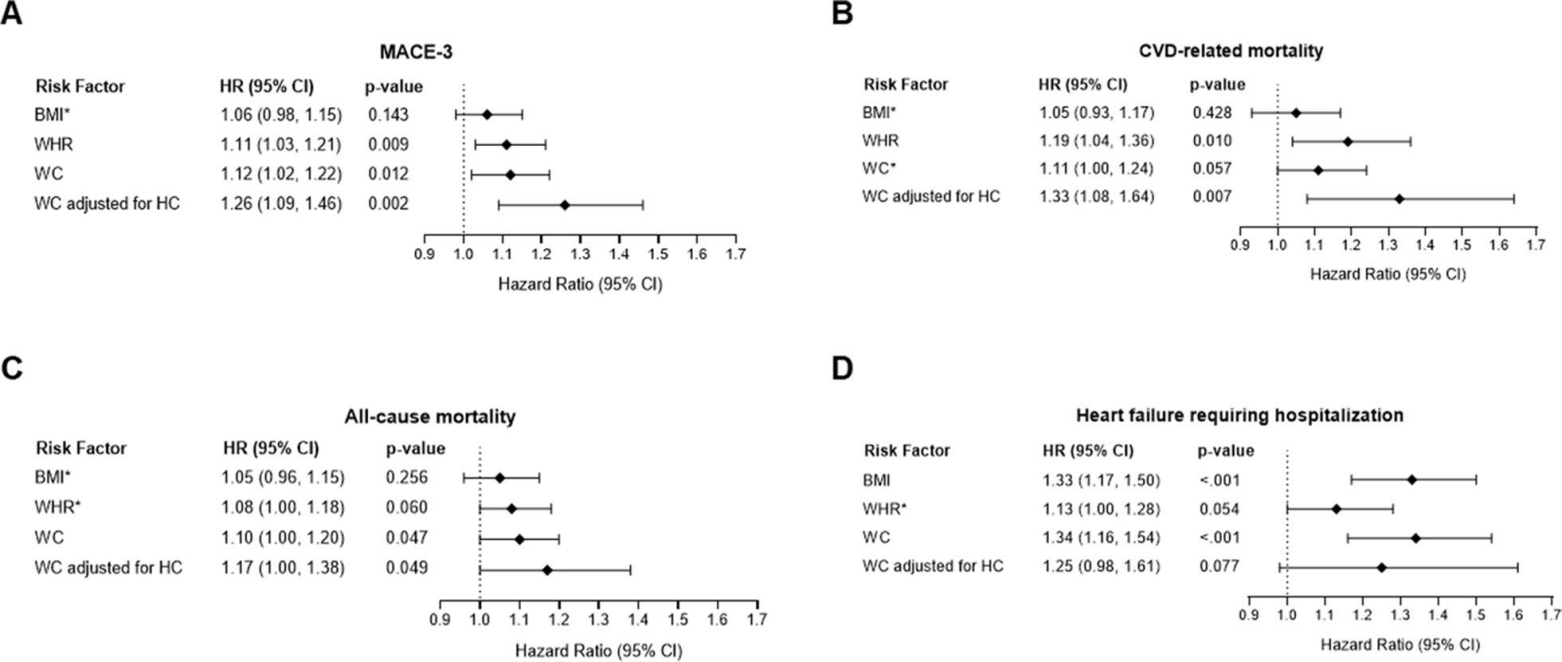
Association of BMI, WHR, WC, and WC adjusted for HC with (**A**) MACE-3, (**B**) CVD-related mortality, (**C**) all-cause mortality, and (**D**) HF requiring hospitalization or urgent care. Results are estimated from Cox proportional hazard regression models. Results are presented per 1 SD increase (WHR 0.08; BMI 5.8 kg/m^2^; WC 13.4 cm; HC 12.7 cm). All obesity measures were evaluated, after adjustment for age and sex (Step 1 of the statistical analysis approach). Those that were significant (p < 0.05) progressed to Step 2 (adjustment for age, sex, and selected baseline factors from the LASSO selection process), otherwise the process stopped after Step 1 (*). *BMI* body mass index, *CI* confidence interval, *CVD* cardiovascular disease, *HC* hip circumference, *HF* heart failure, *HR* hazard ratio; MACE = major adverse cardiovascular events, *SD* standard deviation; *WC* waist circumference, *WHR* waist-to-hip ratio

Additional file 1: Fig. S2 shows the results for all obesity measures after adjustment for age and sex. After adjusting for additional variables identified as significant risk factors for the outcomes using the LASSO selection method and detailed in Additional file 1: Table S1, the relationship between obesity measures and outcomes varied by the outcome (Fig. 1).

For MACE-3, WHR was found to be a significant independent risk factor (HR = 1.11; 95% CI 1.03 to 1.21; p = 0.009), as was WC (HR = 1.12; 95% CI 1.02 to 1.22; p = 0.012) in the fully adjusted model. The analysis of WC adjusted for HC emerged as the strongest risk factor for MACE-3 (HR = 1.26; 95% CI 1.09 to 1.46; p = 0.002). When either BMI, WHR, and WC or BMI, WC, and HC were included together, the resulting multivariable models did not identify any obesity measure (including WC adjusted for HC) as being significantly associated with MACE-3.

For CVD-related mortality, WHR was identified as a significant risk factor (HR = 1.19; 95% CI 1.04 to 1.36; p = 0.010). WC was not significant when included alone (p = 0.057) but became significant when adjusted for HC, with the strongest association of the four measures for CVD-related mortality (HR = 1.33; 95% CI 1.08 to 1.64; p = 0.007). In the model investigating BMI, WHR, and WC together, only WHR was included via the selection process, resulting in the same final model as the respective single model (HR = 1.19; 95%-CI 1.04 to 1.36; p = 0.010). In the model investigating BMI, WC, and HC together, none of the obesity measures were selected. However, when HC was forced into the model, WC was selected, resulting in the same final model as above (HR = 1.33; 95% CI 1.08 to 1.64; p = 0.007).

For all-cause mortality, WC emerged as a significant risk factor (HR = 1.10; 95% CI 1.00 to 1.20; p = 0.047) and remained significant when adjusted for HC, with the largest HR (HR = 1.17; 95% CI 1.00 to 1.38; p = 0.049). In both models investigating a combination of three obesity measures, none of them were included via the selection process. This did not change in the latter model when HC was a forced factor.

BMI was a significant independent risk factor for HF requiring hospitalization (Fig. 1D; HR = 1.33; 95% CI 1.17 to 1.50; p < 0.001). While WC alone was significant (HR = 1.34; 95% CI 1.16 to 1.54; p < 0.001), it became nonsignificant after adjusting for HC (p = 0.077). In both models investigating a combination of three obesity measures, only BMI was included via the selection process, resulting in the same final model as the respective single model (HR = 1.33; 95% CI 1.17 to 1.50; p < 0.001).

In all models no significant interaction with sex was observed.

## Discussion

This post hoc analysis of the placebo group of the REWIND CV Outcomes Trial showed that the anthropometric measures WHR and/or WC, but not BMI, were risk factors for MACE-3, CVD-related mortality, and all-cause mortality in patients with type 2 diabetes and CV risk factors or established CVD. BMI was a significant risk factor only for HF requiring hospitalization. WHR and/or WC were risk factors for all four outcomes, with varying strengths of associations when analyzed in a combination model with other obesity measures. WC adjusted for HC was one of the strongest risk factors for MACE-3, CVD-related mortality, and all-cause mortality, indicating that both WC and HC have independent information pertaining to CV risk which is not completely captured by WHR.

### General adiposity poorly reflects the risk of CV outcomes

While used routinely in clinical practice, increasing BMI is not a reliable universal risk factor for CV-related outcomes in patients with overweight or obesity. Data from the ORIGIN trial showed that obesity, categorized using BMI, had a U-shaped association with mortality and CV outcomes, and patients with overweight and moderate obesity (BMI 25–35 kg/m^2^) had the lowest mortality risk [19]. Similarly, a meta-analysis showed that the BMI category associated with the lowest risk of mortality in patient groups with varying CV risk was the overweight category (BMI 25-29.9 kg/m^2^) [20]. Our results indicate that in the REWIND placebo group, with an inclusion criterion of ≥ 23 kg/m^2^ and a mean baseline BMI of 32 kg/m^2^, BMI was not a significant independent risk factor for MACE-3, CVD-related mortality, or all-cause mortality. BMI was significant for HF requiring hospitalization. The relationship between BMI and HF has been documented previously [21, 22], including in patients with type 2 diabetes [23], and may be explained by the fact that fluid retention is a key contributor to the development of HF [24]. Additionally, it is recommended to use BMI with caution in patients with Asian ancestry, older adults, and muscular adults [25], further limiting its usefulness in the clinical setting. Overall, with the exception of HF, BMI may not be an accurate measure of patients’ risk of cardiovascular outcomes.

### Central adiposity measures as recommended risk factors for CV outcomes

Our results showed that either WHR or WC were risk factors for MACE-3, CVD-related mortality, and all-cause mortality. Given that different measures of obesity indicate general adiposity versus specific areas of fat depots, such as central fat, this may translate to different physiological effects and therefore varied associations with different outcomes. Although reports differ, most studies suggest that central obesity, measured by WHR or WC, is a risk factor for CVD [4], myocardial infarction [6], and CVD-related mortality [5]. Additionally, WC is a principal risk factor for a high metabolic syndrome score [26], and central obesity is associated with an increased risk of HF hospitalization or death in patients with type 1 diabetes [27]and type 2 diabetes [28]. In addition, multivariable Mendelian randomization analyses suggest that the risk of BMI on hospital admission rates is attenuated by WHR [29]. Higher central fat deposition increases the risk for CV events compared to subcutaneous fat deposition which is potentially caused by the impairment of CV mechanics by visceral adipose tissue; data which are captured by WC or WHR measures but not BMI or skinfold thickness [30]. Additionally, fat depots, particularly visceral and ectopic stores, are linked to increased levels of inflammatory mediators such as adipokines, which may drive decreased cardiac function in patients with central obesity anthropometric measures [31]. While some reports suggest that WC, WHR, and waist-to-height ratio can be used to predict all-cause mortality [5], most indicate that there are no differences in risk prediction by central and general obesity measures [8–11]. This divergence from the current results may be due to differences in study populations as the current study investigated patients aged ≥ 50 years with type 2 diabetes. Waist-to-height ratio was not explored in the current study, however, a previous study suggests it may be a better indicator than other central adiposity measurements for evaluating cardio-cerebrovascular events collectively [32]. Although sex has been to shown to play a role in some risk models [12, 13], we did not identify any interaction between sex and any of the obesity factors.

Given the strong evidence that central adiposity can inform patients’ risk of CV events, guidelines should include detail on collecting these measures in addition to weight and BMI. It is increasingly more widely acknowledged that central adiposity can not only contribute to CV risk but also to type 2 diabetes pathology [33]. The American Diabetes Association recommends assessing patients’ weight distribution to guide risk stratification and treatment plans [2] and the American Heart Association (AHA) and American College of Cardiology (ACC) have highlighted the importance of recording patients’ WC as well as BMI [25, 34, 35]. Patients should be individually treated according to both their BMI and WC category. Risk calculators such as the Framingham Risk Score and the ACC ASCVD calculator are valuable tools to assess CV risk and guide treatment strategies and should include weight, height, WC, and HC to fully inform on risk. Due to the heterogenous nature of obesity, one anthropometric measure does not suffice to inform patients’ risk of different CV outcomes.

## Strengths and limitations

This study had several strengths. The REWIND placebo group was a large cohort of patients with type 2 diabetes and CV risk factors or established CVD. The follow-up period was long (median 5.4 years). The REWIND study protocol did not prescribe interventions on body weight or weight change advice. The REWIND trial data provided detailed information such as general and central adiposity in addition to multiple risk factors which are not typically available in other settings.

This study also had limitations. This was a post hoc analysis that was not prespecified. Participants in the REWIND trial had a history of CVD or CV risk factors so results may not be generalizable to patients with no history or risk factors. Likewise, REWIND participants had type 2 diabetes which limits generalisability to other populations. The full spectrum of BMI was unlikely to be represented. Despite multivariable adjustments, some baseline differences may be unaccounted for which limits conclusions. For outcomes other than MACE, the power is low since there were much fewer events. No causal inference can be concluded from the observed associations.

## Conclusions

In a cohort of patients with type 2 diabetes with high risk for CVD, different general and central measures of obesity better reflected patients’ risk of CV events. There was no single obesity measure that was a risk factor for all outcomes (MACE-3, CVD-related or all-cause mortality, or HF requiring hospitalization), however WHR, WC and/or WC adjusted for HC were risk factors for most outcomes. Measuring BMI, WC, and HC collectively may be the most appropriate when assessing the risk of CV events in patients with type 2 diabetes and obesity.

## Supplementary Information

**Additional file 1: Figure S1.** Percentage of participants in the baseline Normal or Obese WC category in each BMI category. **Figure S2.** Association of BMI, WHR, WC, an WC adjusted for HC with (**A**) MACE-3, (**B**) CVD-related mortality, (**C**) all-cause mortality, and (**D**) HF requiring hospitalization or urgent care, minimally adjusted for age and sex (Step 1 of the statistical analysis approach). **Table S1.** Significant baseline characteristics used as additional risk factors to adjust for obesity measures.

## Data Availability

Eli Lilly and Company provides access to all individual participant data collected during the trial, after anonymization, with the exception of pharmacokinetic or genetic data. Data are available to request 6 months after the indication studied has been approved in the US and EU and after primary publication acceptance, whichever is later. No expiration date of data requests is currently set once data are made available. Access is provided after a proposal has been approved by an independent review committee identified for this purpose and after receipt of a signed data sharing agreement. Data and documents, including the study protocol, statistical analysis plan, clinical study report, blank or annotated case report forms, will be provided in a secure data sharing environment. For details on submitting a request, see the instructions provided at www.vivli.org.

## Abbreviations

BMI: Body mass index
CPH: Cox proportional hazards
CI: Confidence intervals
CV: Cardiovascular
CVD: Cardiovascular disease
eGFR: Estimated glomerular filtration rate
HC: Hip circumference
HDL: High-density lipoprotein cholesterol
HF: Heart failure
HR: Hazard ratio
LDL: Low-density lipoprotein cholesterol
MACE: Major adverse cardiovascular events
MI: Myocardial infarction
SD: Standard deviation
UACR: Urine albumin-to-creatinine ratio
WC: Waist circumference
WHR: Waist-to-hip ratio
