# Supplementary material for: General versus central adiposity as risk factors for cardiovascular-related outcomes in a high-risk population with type 2 diabetes: a post hoc analysis of the REWIND trial
Source: Cardiovasc Diabetol. 2023 Mar 10;22:52. doi: 10.1186/s12933-023-01757-z (PMC9999507; doi:10.1186/s12933-023-01757-z)
Supplement: Supplementary file 1 — Additional file 1: Figure S1. Percentage of participants in the baseline Normal or Obese WC category in each BMI category. Figure S2. Association of BMI, WHR, WC, an WC adjusted for HC with (A) MACE-3, (B) CVD-related mortality, (C) all-cause mortality, and (D) HF requiring hospitalization or urgent care, minimally adjusted for age and sex (Step 1 of the statistical analysis approach). Table S1. Significant baseline characteristics used as additional risk factors to adjust for obesity measures. [file 12933_2023_1757_MOESM1_ESM.docx]

**Supplementary Appendix**

**General versus central adiposity as risk factors for cardiovascular-related outcomes in a high-risk population with type 2 diabetes: A post hoc analysis of the REWIND trial**

Franek et al.

**Corresponding author:**

Hong Kan, PhD, MA, MPP

Lilly Corporate Center

Indianapolis, IN 46285

Telephone: +1 317-954-9088

Email: [kan_hongjun@lilly.com](mailto:kan_hongjun@lilly.com)

**Figure S1.** Percentage of participants in the baseline Normal or Obese WC category in each BMI category. Normal WC was defined as: <90 cm for male or <80 cm for female participants in the normal BMI category; <100 cm for male or <90 cm for female participants in the overweight BMI category; <110 cm for male or <105 cm for female participants in the obesity Class I category; and <125 cm for male or <115 cm for female participants in the obesity Class II category. Obese WC was defined as: ≥90 cm for male or ≥80 cm for female participants in the normal BMI category; ≥100 cm for male or ≥90 cm for female participants in the overweight BMI category; ≥110 cm for male or ≥105 cm for female participants in the obesity Class I category; and ≥125 cm for male or ≥115 cm for female participants in the obesity Class II category.

Abbreviations: BMI = body mass index; WC = waist circumference.


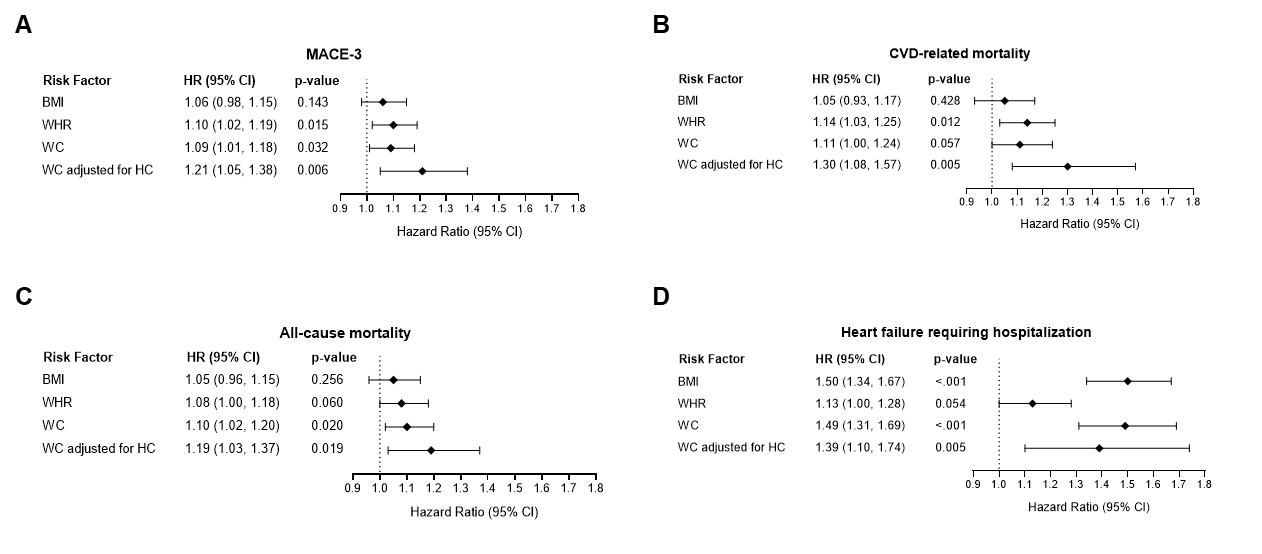


**Figure S2.** Association of BMI, WHR, WC, an WC adjusted for HC with (**A**) MACE-3, (**B**) CVD-related mortality, (**C**) all-cause mortality, and (**D**) HF requiring hospitalization or urgent care, minimally adjusted for age and sex (Step 1 of the statistical analysis approach). Results are estimated from Cox proportional hazard regression models. Results are presented per 1 SD increase (WHR 0.08; BMI 5.8 kg/m²; WC 13.4 cm; HC 12.7 cm).

Abbreviations: BMI = body mass index; CI = confidence interval; CVD = cardiovascular disease; HC = hip circumference; HF = heart failure; HR = hazard ratio; MACE = major adverse cardiovascular events; SD = standard deviation; WC = waist circumference; WHR = waist-to-hip ratio.

**Table S1.** Significant baseline characteristics used as additional risk factors to adjust for obesity measures. Results are from Step 2 of the statistical analysis approach and were used to adjust significant obesity predictors of CV outcomes identified in Step 1. *Factor violated the proportional hazard assumption, therefore no HR or 95% CI has been derived.

|  | **MACE-3** | | | | | | | | | **CVD-related mortality** | | | | | |
| --- | --- | --- | --- | --- | --- | --- | --- | --- | --- | --- | --- | --- | --- | --- | --- |
|  | **WHR** | | | **WC** | | | **WC adjusted HC** | | | **WHR** | | | **WC adjusted HC** | | |
| **Risk factor** | **HR** | **95% CI** | **p value** | **HR** | **95% CI** | **p value** | **HR** | **95% CI** | **p value** | **HR** | **95% CI** | **p value** | **HR** | **95% CI** | **p value** |
| Age (5 year increase) | 1.22 | 1.15, 1.30 | <0.001 | 1.23 | 1.16, 1.31 | <0.001 | 1.23 | 1.15, 1.31 | <0.001 | 1.40 | 1.29, 1.53 | <0.001 | 1.41 | 1.29, 1.54 | <0.001 |
| Sex (male) | 1.06 | 0.87, 1.29 | 0.562 | 1.12 | 0.93, 1.35 | 0.231 | 1.09 | 0.89, 1.35 | 0.401 | 1.18 | 0.88, 1.56 | 0.264 | 1.17 | 0.88, 1.56 | 0.275 |
| Current tobacco use | * | * | <0.001 | * | * | <0.001 | * | * | <0.001 | 1.59 | 1.16, 2.19 | 0.004 | 1.61 | 1.17, 2.21 | 0.003 |
| Past tobacco use | − | − | − | − | − | − | − | − | − | − | − | − | − | − | − |
| Current alcohol consumption | − | − | − | − | − | − | 0.84 | 0.70, 1.02 | 0.075 | 0.65 | 0.50, 0.86 | 0.003 | 0.65 | 0.50, 0.86 | 0.003 |
| UACR (5 mg/mmol increase) | 1.01 | 1.01, 1.02 | <0.001 | 1.01 | 1.01, 1.02 | <0.001 | 1.01 | 1.01, 1.02 | <0.001 | 1.02 | 1.01, 1.02 | <0.001 | 1.02 | 1.01, 1.02 | <0.001 |
| Baseline eGFR | − | − | − | − | − | − | − | − | − | 0.99 | 0.99, 1.00 | 0.002 | 0.99 | 0.99, 1.00 | 0.002 |
| Prior CVD (yes) | 1.51 | 1.15, 1.98 | 0.003 | 1.52 | 1.16, 2.00 | 0.002 | 1.54 | 1.17, 2.03 | 0.002 | 1.65 | 1.20, 2.28 | 0.002 | 1.67 | 1.21, 2.30 | 0.002 |
| History of MI (yes) | 1.53 | 1.18, 1.97 | 0.001 | 1.54 | 1.20, 2.00 | <.001 | 1.52 | 1.18, 1.96 | 0.001 | 1.48 | 1.05, 2.09 | 0.026 | 1.49 | 1.05, 2.10 | 0.025 |
| Ischemic stroke | 1.64 | 1.19, 2.24 | 0.002 | 1.63 | 1.19, 2.23 | 0.003 | 1.58 | 1.15, 2.17 | 0.005 | − | − | − | − | − | − |
| Unstable angina | − | − | − | − | − | − | − | − | − | − | − | − | − | − | − |
| Hospitalization for unstable angina with ECG changes | 1.34 | 1.05, 1.73 | 0.020 | 1.33 | 1.04, 1.71 | 0.025 | 1.33 | 1.04, 1.71 | 0.025 | − | − | − | − | − | − |
| HDL-C (mmol/L) | 0.80 | 0.61, 1.06 | 0.124 | 0.80 | 0.60, 1.06 | 0.113 | 0.84 | 0.63, 1.11 | 0.214 | 0.48 | 0.31, 0.73 | <0.001 | 0.48 | 0.31, 0.73 | <0.001 |
| LDL-C (mmol/L) | 1.20 | 1.11, 1.31 | <0.001 | 1.21 | 1.12, 1.31 | <0.001 | 1.20 | 1.11, 1.30 | <0.001 | 1.26 | 1.12, 1.41 | <0.001 | 1.26 | 1.13, 1.41 | <0.001 |
| Systolic blood pressure (5 mmHg increase) | 1.06 | 1.03, 1.08 | <0.001 | 1.05 | 1.03, 1.08 | <0.001 | 1.05 | 1.03, 1.08 | <0.001 | 1.05 | 1.02, 1.09 | 0.004 | 1.05 | 1.02, 1.09 | 0.004 |
| Anticoagulant agents | − | − | − | − | − | − | − | − | − | − | − | − | − | − | − |
| Antithrombotic agents | − | − | − | − | − | − | − | − | − | − | − | − | − | − | − |
| HC (1 SD/12.7 cm increase) | − | − | − | − | − | − | 0.87 | 0.75, 1.01 | 0.058 | − | − | − | 0.82 | 0.66, 1.01 | 0.059 |
|  | **All-cause mortality** | | | | | | **HF requiring hospitalization** | | | | | | | | |
|  | **WHR** | | | **WC adjusted HC** | | | **BMI** | | | **WC** | | | **WC adjusted HC** | | |
| **Risk factor** | **HR** | **95% CI** | **p value** | **HR** | **95% CI** | **p value** | **HR** | **95% CI** | **p value** | **HR** | **95% CI** | **p value** | **HR** | **95% CI** | **p value** |
| Age (5 year increase) | 1.38 | 1.29, 1.47 | <0.001 | 1.37 | 1.28, 1.47 | <0.001 | 1.13 | 1.02, 1.26 | 0.017 | 1.12 | 1.01, 1.24 | 0.034 | 1.12 | 1.01, 1.24 | 0.031 |
| Sex (male) | 1.15 | 0.94, 1.41 | 0.166 | 1.10 | 0.89, 1.37 | 0.376 | 1.32 | 0.94, 1.85 | 0.109 | 1.09 | 0.78, 1.52 | 0.606 | 1.15 | 0.79, 1.68 | 0.465 |
| Current tobacco use | 1.44 | 1.12, 1.84 | 0.004 | 1.43 | 1.11, 1.83 | 0.005 | − | − | − | − | − | − | − | − | − |
| Past tobacco use | − | − | − | − | − | − | 1.38 | 1.00, 1.90 | 0.051 | 1.38 | 1.00, 1.90 | 0.050 | 1.37 | 1.00, 1.89 | 0.053 |
| Current alcohol consumption | − | − | − | − | − | − | − | − | − | − | − | − | − | − | − |
| UACR (5 mg/mmol increase) | 1.02 | 1.01, 1.02 | <0.001 | 1.02 | 1.01, 1.02 | <0.001 | 1.00 | 1.00, 1.01 | 0.182 | 1.00 | 1.00, 1.01 | 0.169 | 1.00 | 1.00, 1.01 | 0.178 |
| Baseline eGFR | 0.99 | 0.99, 0.99 | <0.001 | 0.99 | 0.99, 0.99 | <0.001 | 1.00 | 0.99, 1.00 | 0.242 | 1.00 | 0.99, 1.00 | 0.188 | 1.00 | 0.99, 1.00 | 0.207 |
| Prior CVD (yes) | 1.36 | 1.04, 1.79 | 0.027 | 1.37 | 1.04, 1.81 | 0.024 | − | − | − | − | − | − | − | − | − |
| History of MI (yes) | 1.42 | 1.06, 1.89 | 0.017 | 1.41 | 1.06, 1.88 | 0.019 | 1.53 | 1.11, 2.13 | 0.010 | 1.51 | 1.09, 2.09 | 0.014 | 1.52 | 1.09, 2.12 | 0.012 |
| Ischemic stroke | 1.79 | 1.27, 2.51 | <0.001 | 1.78 | 1.27, 2.50 | <0.001 | − | − | − | − | − | − | − | − | − |
| Unstable angina | − | − | − | − | − | − | 1.35 | 0.85, 2.15 | 0.199 | 1.34 | 0.84, 2.13 | 0.215 | 1.33 | 0.84, 2.12 | 0.224 |
| Hospitalization for unstable angina with ECG changes | − | − | − | − | − | − | − | − | − | − | − | − | − | − | − |
| HDL-C (mmol/L) | 0.56 | 0.41, 0.76 | <0.001 | 0.57 | 0.41, 0.77 | <0.001 | − | − | − | − | − | − | − | − | − |
| LDL-C (mmol/L) | 1.17 | 1.07, 1.27 | <0.001 | 1.16 | 1.06, 1.27 | <0.001 | − | − | − | − | − | − | − | − | − |
| Systolic blood pressure (5 mmHg increase) | 1.03 | 1.00, 1.06 | 0.037 | 1.03 | 1.00, 1.06 | 0.036 | − | − | − | − | − | − | − | − | − |
| Anticoagulant agents | − | − | − | − | − | − | * | * | 0.023 | * | * | 0.020 | * | * | 0.020 |
| Antithrombotic agents | − | − | − | − | − | − | * | * | <0.001 | * | * | <0.001 | * | * | <0.001 |
| HC (1 SD/12.7 cm increase) | − | − | − | 0.92 | 0.79, 1.08 | 0.315 | − | − | − | − | − | − | 1.08 | 0.84, 1.39 | 0.550 |

Abbreviations: CI = confidence interval; CVD = cardiovascular disease; ECG = electrocardiogram; eGFR = estimated glomerular filtration rate; HC = hip circumference; HDL = high-density lipoprotein cholesterol; HR = hazard ratio; LDL = low-density lipoprotein cholesterol; MACE = major adverse cardiovascular events; MI = myocardial infarction; SD = standard deviation; UACR = urine albumin-to-creatinine ratio; WC = waist circumference; WHR = waist-to-hip ratio
